# Family Cohesion and Stress Consequences Among Chinese College Students During COVID-19 Pandemic: A Moderated Mediation Model

**DOI:** 10.3389/fpubh.2021.703899

**Published:** 2021-07-15

**Authors:** Yadi Zeng, Baojuan Ye, Yanzhen Zhang, Qiang Yang

**Affiliations:** ^1^Center of Preschool Education, Center of Mental Health Education and Research, School of Psychology, Jiangxi Normal University, Nanchang, China; ^2^Department of Psychology, University of California, Riverside, Riverside, CA, United States; ^3^Center of Preschool Education, School of Education, Jiangxi Normal University, Nanchang, China

**Keywords:** family cohesion, fear of COVID-19, cognitive empathy, affective empathy, stress consequences, Chinese college students

## Abstract

Family plays a pivotal role in individuals' mental health. During the COVID-19 epidemic, people were being quarantined at home to prevent the further spread of the virus. Therefore, the influence of family on individuals is more significant than usual. It is reasonable to assume that family cohesion can effectively alleviate the stress consequences during the COVID-19 epidemic. In the present study, a moderated mediation model was constructed to examine the mechanisms underlying the association between family cohesion and stress consequences among Chinese college students. A large sample of Chinese college students (*N* = 1,254, *M*_age_ = 19.85, *SD*_age_ = 1.29) participated in the study. Results indicated that family cohesion was negatively related to stress consequences. Fear of COVID-19 partially mediated the link between family cohesion and stress consequences. Excessive affective empathy reported by participants served to aggravate the relation between fear of COVID-19 and stress consequences. The study helps us understand how internal and external factors affect individual mental health that provides meaningful implications for promoting mental health.

## Introduction

Globally, as of June 9, 2021, COVID-19 has lasted over a year which has resulted in over 173 million confirmed cases of COVID-19, including over 3.7 million deaths (1). Undoubtedly, COVID-19 poses a threat to individuals' physical and mental health and seriously disrupts their normal life (2). During the COVID-19 epidemic, most people show different degrees of anxiety (66.9%), worry (71.7%), and fear (58.2%) (3) and 40% of people suffer from insomnia (4). In addition, college students have higher learning burnout during the COVID-19 epidemic (5). The stress-induced consequences stemming from the COVID-19 pandemic can be regarded as stress consequences, which refer to the individual's physical and psychological stress reaction caused by external pressure (6, 7). Given the risks of the COVID-19 epidemic, it's highly imperative to design effective social interventions of stress consequences amongst college students who are at the developmental stage from adolescence to adulthood.

In order to avoid the further spread of COVID-19, citizens were advised to stay at home as much as possible to avoid non-essential contact with others. Because of home quarantine, we contact family members most frequently. Family cohesion is closely correlated with depression, insomnia, learning burnout, and anxiety (8–10), which are the manifestation of stress consequences. Thus, family cohesion may be also negatively correlated with stress consequences. There is no direct research to prove the relationship between family cohesion and stress consequences, nonetheless, we can conjecture that from previous studies. Thus, we explored and tested whether family cohesion was significantly related to stress consequences among Chinese college students during the COVID-19 pandemic and the underlying mechanisms in the relationship.

### Family Cohesion and Stress Consequences

Family is an important environment for individual healthy development (11). Family cohesion comprises the emotional bonding between family members and the degree of autonomy experienced by individuals within the family system (12, 13). In the family, the primary goal is to fulfill various tasks including crisis tasks according to family process model theory (14). Some empirical studies have shown that family cohesion can alleviate the individual psychological problems contributing to mental health (15–17). According to the main-effect model (18), friendly relationships with the family can provide support for individuals under stress and promote healthy physical and mental development. The higher the family cohesion, the more support and help the individual can get from others, which can help the individual reduce the negative effects of stress. During the outbreak of COVID-19, individuals may suffer from psychological problems (e.g., depression), physical symptoms (e.g., insomnia), and behavior of weariness due to the pressure brought by the COVID-19 epidemic, which are the manifestation of the stress consequences (6). Accordingly, we assume that family cohesion may be negatively related to stress consequences.

### Fear of COVID-19 as a Mediator

Likely, the relation between family cohesion and stress consequence is not just a simple and direct one. Studies have shown that there are mediating variables between family cohesion and stress consequences [e.g., insomnia; (19)]. Fear of COVID-19 which received widespread attention is noteworthy (20–22). Triggered by the novelty and uncertainty of COVID-19, fear of COVID-19 is a negative emotion, especially revealing in physical aspect (e.g., fear of infection), possibly leading to maladaptation [e.g., depression, anxiety; (23–25)]. Possibly, family cohesion can alleviate stress consequences by relieving the fear of COVID-19. Although not yet tested, it is reasonable to expect that fear of COVID-19 acts as a mediator between family cohesion and stress consequences.

Family cohesion is an important protective factor which is negatively related to negative emotion (26–29). As expected, the study during the COVID-19 epidemic period also has found a significant negative relationship between family cohesion and fear of COVID-19 (30). In families with a high level of cohesion, harmonious family communication can convey a sense of support and security, which can help individuals ease their fear of strange things (31). Fear of COVID-19 is a naturally occurring negative emotion due to the strangeness of the COVID-19. So, family cohesion may be negatively related to fear of COVID-19. According to the broaden-and-build theory, the sense of pleasure brought by family can alleviate the negative emotions of individuals (32). During the COVID-19 epidemic period, with higher family cohesion, the communication between individuals and their families is more pleasant and harmonious. The pleasure brought by the family helps to alleviate their fear. Besides, intimacy and love among family members can broaden the individual's thoughts (33) which can avoid paying too much attention to negative information of the COVID-19 epidemic increasing their negative emotion (e.g., fear). Also, the higher level of the family cohesion, the higher the frequency of communication between individuals and their families. It can be said that the current Chinese college students who suffer from the lack of psychological preparation and epidemic prevention experience have never experienced infectious disease with such a large scale and strong infectivity. Communicating with parents is also an effective way to gain experience for coping with the COVID-19 actively, thereby contributing to alleviating fear. Thus, family cohesion may be negatively related to fear of COVID-19.

Negative emotions may be closely related to stress consequences. Studies have shown that basic emotions (e.g., fear) are the basis for the development of complex emotions [e.g., depression; (34)]. An empirical study of adolescent survivors of the Wenchuan earthquake also has shown that fear is an important risk factor for depression (35). What's more, fear of COVID-19 is positively related to depression, anxiety (36–39), which are the manifestation of the stress consequences (6). Thus, as a basic emotion, fear of COVID-19 may be an important predictor of stress consequences which includes depression, anxiety. During the COVID-19 epidemic, it is quite normal for individuals to fear their families' safety as well as their own. If such feelings cannot be timely and effectively alleviated, the long-term fear is likely to lead to anxiety, depression, and physical disorders (stress consequences). Based on the above findings, we assume that fear of COVID-19 is positively associated with stress consequences. Thus, fear of COVID-19 may mediate the relation between family cohesion and stress consequences.

### Empathy as a Moderator

Although family cohesion may decrease the impact of stress consequences through the mediating role of fear of COVID-19, not all individuals with higher fear of COVID-19 will equally perceive stress consequences. So, it is necessary to explore potential moderating variables that may influence the relation between fear of COVID-19 and stress consequences. Empathy refers to an affective state that is elicited by observing or imagining the other's affective state, is similar to the other's emotional state and is caused by the other's emotional state (40). We often regarded empathy as a positive trait, promoting benign effect (41). However, from a more comprehensive perspective, the results of empathy include both positive and negative effects. In addition, some studies have shown that empathy is a multi-dimensional construct that includes both cognitive and affective empathy (42), which have different effects on stress consequences [e.g., depression, anxiety; (43)].

Affective empathy refers to our ability to experience an emotion similar to that of another person, even though the event that causes the emotion doesn't directly happen to us (44). The study showed that there was a positive association between affective empathy and anxiety (45) which was an obvious manifestation of stress consequences (6). Not surprisingly, affective empathy is also proved to be a risk factor for mental health in previous studies (46, 47). Wright et al. who explored the moderating effect of affective empathy found that affective empathy aggravated the adverse effect of other risk factors on depression (48). Thus, excessive affective empathy may be a moderator increasing the negative effects of fear of COVID-19 on stress consequences. Specifically, the impact of fear of COVID-19 on stress consequences may be stronger for college students with higher affective empathy.

Cognitive empathy refers to the recognition, understanding, and mentalizing of others' emotions (49). Evidence showed that cognitive empathy appeared positive for psychological health (16). Based on the risk buffering model (50), protective factors may reduce the negative impact of risk factors. Fear of COVID-19 as an emotional factor may hasten more serious stress consequences, which can be regarded as a risk factor promoting stress consequences. Cognitive empathy negatively correlated with depression and anxiety (51). Cognitive empathy can be considered as a protective factor to buffer the adverse effects of fear of COVID-19 on stress consequences. Specifically, the impact of fear of COVID-19 on stress consequences may be weaker for college students who report higher cognitive empathy.

### The Present Study

**Figure 1:**
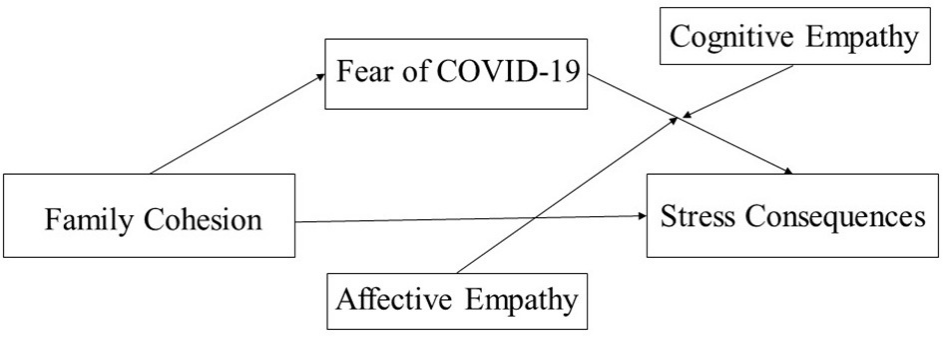
The proposed moderated mediation model.

Based on the literature review, we proposed the following hypotheses, as (Figure 1) shows:

Hypothesis 1. Family cohesion is negatively related to stress consequences.

Hypothesis 2. Fear of COVID-19 will mediate the relationship between family cohesion and stress consequences.

Hypothesis 3. (a) Excessive affective empathy will moderate the relationship between fear of COVID-19 and stress consequences. (b) Cognitive empathy will moderate the relationship between fear of COVID-19 and stress consequences (Figure 1).

## Method

### Participants

The study was approved by the Research Ethics Committee of the first author's institution and hosted on Survey Star (Changsha Ranxing Science and Technology, Shanghai, China) from March 16 to March 23, 2020. We obtained consent from all participating college students before the data collection. A total of 1,254 students (*M*_age_ = 19.85, *SD*_age_ = 1.29, Range_age_ = 18–25, 66% female) anonymously completed the survey on measures including demographic variables, family cohesion, fear of COVID-19, empathy, and stress consequences. Among the total sample, 556(44.3%) were first years, 530 (42.3%) were second years, 116 (9.3%) were third years, and 52 (4.1%) were fourth years.

### Measures

#### Family Cohesion

Family cohesion was measured by the cohesion dimension of the family adaptability and cohesion evaluation scale (13, 52). With higher total scores indicating higher levels of family cohesion, the scale consisted of 16 items (e.g., “The relationship between family members is very close”) on a 5-point scale (1 = never, 5 = always), α = 0.820. Good reliability and validity of the family cohesion scale have been proved among Chinese participants (53–56). Confirmatory factor analysis (CFA) suggested that the model fit the data well: CFI = 0.998, TLI = 0.991, RMSEA = 0.053, 90%CI = [0.012, 0.106], SRMR = 0.008.

#### Fear of COVID-19

Fear of COVID-19 was measured by the fear of COVID-19 scale (30). Participants rated 9 items (e.g., “I worry about being infected by others”) on a five-point scale (1 = never, 5 = always), α = 0.887. Higher scores indicate a higher level of fear of COVID-19. Good reliability and validity of the fear of the COVID-19 scale have been proved among Chinese participants (30). Confirmatory factor analysis (CFA) suggested that the model fit the data well: CFI = 0.994, TLI = 0.990, RMSEA = 0.038, 90%CI = [0.026, 0.049], SRMR = 0.019.

#### Empathy

Empathy was measured by the basic empathy scale (BES) (57, 58). Participants rated 20 items (e.g., “I am easily affected by others' emotions”) on a five-point scale (1 = strongly disagree, 5 = strongly agree) assessing two dimensions of cognitive empathy (9 items) and affective empathy (11 items). Besides, the total score will be calculated after the items (8 items) are scored in reverse. Higher scores indicate a higher level of empathy. Good reliability and validity of the basic empathy scale have been proved among Chinese participants (59). In this study, Cronbach's α for cognitive empathy was 0.784, and Cronbach's α for affective empathy was 0.737. Confirmatory factor analysis (CFA) suggested that the model fit the data well: CFI = 0.998, TLI = 0.991, RMSEA = 0.035, 90%CI = [0.000, 0.074], SRMR = 0.009.

#### Stress Consequences

Stress consequences were measured by the stress consequences scale (7), α = 0.885. Participants rated 17 items (e.g., “You may feel pain in some parts of your body, such as your head or chest”) on a five-point scale (1 = strongly disagree, 5 = strongly agree), assessing three dimensions of behavioral symptoms (5 items), psychological symptoms (6 items), and physical symptoms (3 items). Besides, the total score will be calculated after the items (3 items) are scored in reverse. The higher the score, the stronger the stress consequences. Good reliability and validity of the stress consequences scale have been proved among Chinese participants (7). Confirmatory factor analysis (CFA) suggested that the model fit the data well: CFI = 0.968, TLI = 0.958, RMSEA = 0.054, 90%CI = [0.049, 0.059], SRMR = 0.029.

### Procedure

The research was hosted on Survey Star (Changsha Ranxing Science and Technology, Shanghai, China) and participants were recruited electronically from March 16 to March 23, 2020, when the majority of the population was home isolated due to COVID-19. Participants anonymously completed the tests after informed consent was obtained from the schools, teachers, and participants. Also, participation in this study was entirely voluntary, and thus no compensation was given to participants.

### Data Analysis

Firstly, descriptive statistics and Pearson correlations were calculated among the study variables. Secondly, the PROCESS macro for SPSS (Model 4) was applied to examine the mediating effect of fear of COVID-19 (60). Thirdly, the PROCESS macro (Model 16) was applied to examine the moderating effect of empathy on the indirect links between family cohesion and stress consequences. In the meanwhile, the demographic variables (gender, grade) were controlled when we examined the mediating effect and moderating effect. The bootstrap confidence intervals (CIs) determine whether the effects in Model 4 and Model 16 are significantly based on 5,000 random samples (60). An effect is regarded as significant if the CIs do not include zero. All study variables were standardized in Model 4 and Model 16 before data analyses.

## Result

### Preliminary Analyses

Table 1 showed means, *SD*s, and Pearson correlations for the study variables. As the results showed, family cohesion was negatively correlated with fear of COVID-19 and stress consequences and positively correlated with cognitive empathy. In addition, fear of COVID-19 was positively correlated with affective empathy and stress consequences and negatively correlated with cognitive empathy. Stress consequences were negatively correlated with cognitive empathy and positively correlated with affective empathy.

**Table 1:** Means, standard deviations, and correlations of the main study variables.

	***M***	***SD***	**1**	**2**	**3**	**4**	**5**
1. Family cohesion	4.192	0.622	–				
2. Fear of COVID-19	1.864	0.591	−0.364^***^	–			
3. Cognitive empathy	3.806	0.505	0.274^***^	−0.099^***^	–		
4. Affective empathy	3.497	0.510	−0.008	0.208^***^	0.374^***^	–	
5. Stress consequences	1.850	0.586	−0.499^***^	0.541^***^	−0.078^**^	0.211^***^	–

*N = 1,254;*

** *p < 0.01 and*

*** *p < 0.001*.

### Testing for Mediation Effect

The result showed that family cohesion was negatively correlated with stress consequences supporting Hypothesis 1 (β = −0.480, *t* = −19.945, *p* < 0.001, 95%CI = [−0.527, −0.433]). In Hypothesis 2, we assumed that fear of COVID-19 would mediate the relationship between family cohesion and stress consequences. This hypothesis was tested with Model 4 of the PROCESS macro (50). As Table 2 showed, family cohesion was negatively associated with fear of COVID-19 (β = −0.341, *t* = −13.308, *p* < 0.001, 95%CI = [−0.392, −0.291]), which in turn was positively related to stress consequences (β = 0.387, *t* = 15.974, *p* < 0.001, 95%CI = [0.339, 0.434]). In the meantime, the negative direct association between family cohesion and stress consequences remained significant. The result supported Hypothesis 2. Fear of COVID-19 partially mediated the relationship between family cohesion and stress consequences (indirect effect = −0.132, *SE* = 0.012, 95%CI = [−0.156, −0.110]). The mediation effect accounted for 27.5% of the total effect of family cohesion and stress consequences.

**Table 2:** Testing the mediation effect and moderated mediation effect of family cohesion on stress consequences.

**Predictors**	**Model 1 (FOC)**		**Model 2 (SC)**		**Model 3 (SC)**	
	**β (95%CI)**	***t***	**β (95%CI)**	***t***	**β (95%CI)**	***t***
Gender	−0.178 (−0.284, −0.073)	−3.311^**^	−0.053 (−0.144, 0.037)	−1.154	0.010 (−0.083, 0.104)	0.219
Grade	0.280 (0.216, 0.343)	8.655^***^	0.124 (0.068, 0.180)	4.344^***^	0.103 (0.048, 0.158)	3.652^***^
FC	−0.341 (−0.392, −0.291)	−13.308^***^	−0.348 (−0.394, −0.302)	−14.849^***^	−0.357 (−0.404, −0.310)	−14.957^***^
FOC			0.387 (0.339, 0.434)	15.974^***^	0.361 (0.313, 0.409)	14.767^***^
CE					−0.002 (−0.051, 0.047)	−0.083
FOC × CE					−0.013 (−0.060, 0.034)	−0.557
AE					0.132 (0.083, 0.182)	5.296^***^
FOC × AE					0.098 (0.053, 0.144)	4.264^***^
*R*^2^	0.190		0.408		0.430	
*F*	97.721^***^		215.043^***^		117.549^***^	

*N = 1,254; FC, family cohesion; FOC, fear of COVID-19; CE, cognitive empathy; AE, affective empathy; SC, stress consequences; Gender was dummy coded such that 0 = female and 1 = male; Grade was dummy coded such that 0 = first year, 1 = second year, 2 = third year, 3 = fourth year;*

** *p < 0.01 and*

*** *p < 0.001*.

### Moderated Mediation Effect Analysis

**Figure 2:**
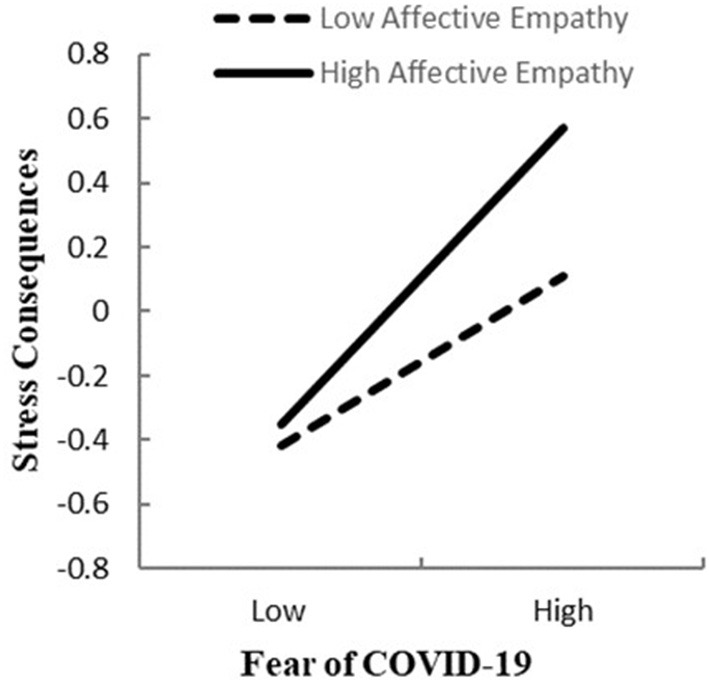
Association between fear of COVID-19 and stress consequences at higher and lower levels of affective empathy.

To test the moderated mediation model, we used Model 16 of the SPSS macro-PROCESS compiled by Masten (50). The results of the empathy moderation test were shown in Table 2. The product (interaction term) of fear of COVID-19 and cognitive empathy didn't have a significant predictive effect on stress consequences (β = −0.013, *t* = −0.557, *p* =0.578, 95%CI = [−0.060, 0.034]). The result did not support Hypothesis 3b. The product (interaction term) of fear of COVID-19 and affective empathy had a significant predictive effect on stress consequences (β = 0.098, *t* = 4.264, *p* < 0.001, 95%CI = [0.053, 0.144]). The result supported Hypothesis 3a. In order to further portray the interaction, we conducted simple slope plots and calculated beta coefficients at −1*SD* and+1*SD* from the mean of affective empathy (Figure 2). The result of simple slope tests showed that for college students with a higher level of affective empathy, the influence of fear of COVID-19 on stress consequences had a steeper slope, meaning it was statistically significant (β_*simple*_ = 0.459, *p* < 0.001, 95%CI = [0.395, 0.523]). For college students with a lower level of affective empathy, the influence of fear of COVID-19 on stress consequences was positively and statistically significant (β_*simple*_ =0.263, *p* < 0.001, 95%CI = [0.194, 0.330]).

The bias-corrected percentile bootstrap analysis further indicated that the indirect effect of family cohesion on stress consequences through fear of COVID-19 was moderated by affective empathy. Particularly, for college students low in affective empathy, the indirect effect of family cohesion on stress consequences through fear of COVID-19 was significant, *b* = −0.090, *SE* = 0.014, 95% CI boot = [−0.117, −0.063]. The indirect effect was also significant for college students with high affective empathy, but stronger, *b* = −0.157, *SE* = 0.016, 95% CI boot = [−0.190, −0.125]. Results indicated that fear of COVID-19 mediated the effect of family cohesion on stress consequences, and affective empathy strengthened the mediating effect of fear of COVID-19 as well.

## Discussion

A moderated mediation model was tested in our study to analyze the mechanisms underlying the association between family cohesion and stress consequences. The result showed that family cohesion was negatively related to stress consequences. Additionally, our findings contributed to the literature by testing a moderated mediation model, showing that fear of COVID-19 was a mediator between family cohesion and stress consequences, and the relation between fear of COVID-19 and stress consequences was moderated by affective empathy. The results help to understand the psychological processes of how family cohesion may lead to less serious stress consequences among Chinese college students.

### The Relationship Between Family Cohesion and Stress Consequences

A significant negative association between family cohesion and stress consequences was found which supported the previous studies on family factors and mental health (61–63). As posited by the cognitive theory of stress and coping, an individual experiences stress when the environment's external demands exceed the individual's internal adaptive capacity (64). As an original environment and living environment of physical and mental growth, family plays an important role when individuals face stress especially for college students who suffer from the lack of necessary life experience and skills to cope with novel problems and negative emotions (65). On the one hand, family cohesion can promote internal resources (e.g., resilience) which is vital to coping with stress (66, 67). Previous studies have shown that individuals with high family cohesion have stronger abilities to regulate negative emotion (68) which can decrease depression, anxiety, and learning burnout which are all manifestations of stress consequences (69–71). On the other hand, family cohesion is the emotional bond connecting family members that compels family members to engage with each other (72). Communicating amicably with family members can help individuals alleviate the stress consequences and promote mental health by releasing individual negative emotions and reaping more experience in coping with problems (73). During the COVID-19 epidemic, people with high family cohesion can deal with stress consequences via better ability for regulation by themselves and get more useful ways from the family to release stress to avoid stress consequences.

### The Mediating Role of Fear of COVID-19

The mediating role of fear of COVID-19 between family cohesion and stress consequences was also tested in the present study. In our study, family cohesion buffered the fear of COVID-19, which in turn was positively related to stress consequences. The first path, wherein family cohesion was negatively related to fear of COVID-19 was consistent with a prior study on family and negative emotion (74). A comfortable and intimate family atmosphere encourages individuals to express their real emotions and feelings (75, 76). A good parent-child relationship has more emotional communication, which can help parents more easily identify children's emotional cues and respond supportively (77). Under the COVID-19 epidemic situation, individuals in families with a high level of cohesion are more likely to confide their fear about COVID-19 and terrible outcomes (e.g., infection and death) to their parents. As a useful way to release emotions (78), expressing emotion clearly to parents can help alleviate fear and avoid deterioration. At the same time, the cohesion among family members can also help parents to detect changes in children's moods. If children are so fearful of COVID-19, parents can provide support to them in time to help them cope with fear better.

The second path of the mediation model, wherein fear of COVID-19 was positively related to stress consequences was consistent with previous studies on stress consequences [e.g., depression; (79)]. As negative emotion, fear of COVID-19 may narrow the scope of attention and thinking action (26). The fear will cause attention bias that people pay more attention to the related negative information aggravating the feelings of helplessness and fear, which may promote the generation and aggravation of the complex emotion (e.g., depression, anxiety), which are all manifestations of stress consequences. Thus, fear of COVID-19 may intensify stress consequences. While fear of COVID-19 was a mechanism that mediated the relation between family cohesion and stress consequences, however, the remaining direct and negative effect suggests that family cohesion still independently affects stress consequences. According to the results, we should pay special attention to the important role of family cohesion which includes two non-ignorable aspects: dispositional and daily family cohesion, both of which are closely related to mental health (80). Therefore, the daily communication and emotional expression of family members play a positive role in helping individuals cope with stress, negative emotions, thereby improving mental health.

### The Moderating Role of Empathy

The results further revealed that excessive affective empathy moderated the path between fear of COVID-19 and stress consequences. As expected, the association between fear of COVID-19 and stress consequences was stronger for college students who reported a higher level of affective empathy. For college students who reported low affective empathy, the relation between fear of COVID-19 and stress consequences was weaker. When others experience difficulties, feeling their experiences may lead to emotional infection and common pain (81), which could be a self-oriented response to others leading to pressure and even negative results (e.g., depression, anxiety) to the perceiver (82). With the help of social media (e.g., microblog), although we were quarantined at home during the COVID-19 epidemic, we could know that many people were suffering from illness. Due to the long incubation period and high infectivity of COVID-19, people would still be nervous and worried about their lives and the safety of their families even if they were quarantined at home. For individuals with high levels of affective empathy, it is easier for them to imagine themselves in the same situation (83) and even possibly regard the painful experiences of others and their family members as the possibility of their own future lives. The perception of other people's pain can aggravate the effect of fear of COVID-19 on stress consequences. The higher the level of affective empathy, the stronger the perception of other people's pain, which will aggravate the impact of fear of COVID-19 on stress consequences. Therefore, we need to pay attention to the individuals with a high level of affective empathy and try to help them decrease the level of affective empathy. For individuals with a lower level of affective empathy, they feel less pain than the people in the epidemic area, which induces a relatively less negative impact on their own emotions, alleviating the impact of fear of COVID-19 on the stress consequences. In addition, the relation between fear of COVID-19 and stress consequences was still significant at a low level of affective empathy. Thus, having a low level of affective empathy does not necessarily negate or reverse the effect entirely. Fear of COVID-19 remains a strong antecedent of stress consequences.

Besides, cognitive empathy didn't moderate the relation between fear of COVID-19 and stress consequences which overturned our hypothesis. Previous studies show that cognitive empathy is related to executive function (84, 85), especially inhibitory control, which may help us to inhibit emotional contagion to regulate our concern about others when we empathize with others (86). Studies have shown that the inhibitory control of individuals would be reduced under negative emotions (87–89). Under the common negative emotional atmosphere of COVID-19, inhibitory control may generally hinder the function of cognitive empathy. Thus, cognitive empathy can't moderate the relation between fear of COVID-19 and stress consequences.

### Limitations and Future Directions

However, several limitations should be noted. First, the present study was cross-sectional, and causality cannot be inferred. Future studies may design longitudinal studies to confirm the causal hypotheses in this study. Second, all measures included in this study were self-report. Future studies may try to collect data from multiple informants (e.g., family members) to deepen the current findings. Third, the sample used in this study is entirely Chinese college students, limiting the extent to which the results of the current study can be generalized across cultures. Further investigation is still needed to test the current hypotheses across cultures.

Despite these limitations, the current study has several theoretical and practical contributions. In terms of theoretical significance, this study further extends previous research by proposing a mediating role of fear of COVID-19 and the moderating role of affective empathy. This will contribute to a better understanding of the importance of family cohesion for alleviating stress consequences and the different functions of cognitive empathy and affective empathy. From a practical perspective, our study may provide useful insights into how social and familial interventions may be designed to reduce college students' stress consequences during a pandemic. It has always been important for the government and schools to monitor and measure the emotional state and mental health of students. Without exception, even during the COVID-19 epidemic, family cohesion remains an important factor that is beneficial to their physical and mental health. College students are supposed to friendly interact with their families more, which can help individuals rationally view the epidemic, regulate their own negative emotions, and reduce fear and stress consequences. Society, school, and family should help students in coping strategies and guide them to adopt positive and effective ways to regulate negative emotions, such as cognitive reappraisal. Also, particularly during the COVID-19 epidemic, news implicates the spread of public empathy, which should be followed characteristics of the development stage of COVID-19, avoid causing extreme negative emotion and empathy (90). For example, reports should be rational and objective to stabilize the public emotion in the early stage of COVID-19. To avoid the harm of excessive affective empathy, authorities can guide individuals to take practical actions (such as donations) to help the critical epidemic area, so as not to overindulge in other people's suffering emotionally.

## Conclusion

Results have shown that fear of COVID-19 serves as one potential mechanism between family cohesion and stress consequences. Moreover, the significant moderation effect of affective empathy warrants further examination of how excessive affective empathy can be detrimental to one's health. This study may give us some advice about how to alleviate stress consequences when we face difficulty (e.g., epidemic).

## Data Availability Statement

The original contributions presented in the study are included in the article/supplementary material, further inquiries can be directed to the corresponding author/s.

## Author Contributions

YZe, BY, and YZh designed the study. YZe collected the data. BY and YZh analyzed the data and conceptualized the models. QY supervised the project. All authors have seen and approved the manuscript and wrote and revised the manuscript.

## Conflict of Interest

The authors declare that the research was conducted in the absence of any commercial or financial relationships that could be construed as a potential conflict of interest.
